# Assessing the psychometric properties and validating the Portuguese version of the INSPIRE measure of staff support for personal recovery in Portugal

**DOI:** 10.1186/s12888-025-07060-3

**Published:** 2025-07-01

**Authors:** Manuela Silva, Deborah Oyine Aluh, Ana Lourenço, Beatriz Resende, Francisco Agostinho, João Bessa Rodrigues, João Pedro Azenha, João Revez Lopes, Marta Ribeiro, Maria João Heitor, Joaquim Gago, Graça Cardoso

**Affiliations:** 1 Lisbon Institute of Global Mental Health, Comprehensive Health Research Centre, Nova University of Lisbon, Lisbon, Portugal; 2 Department of Psychiatry and Mental Health, Unidade Local de Saúde Santa Maria, Lisboa, Portugal; 3 Department of Clinical Pharmacy and Pharmacy Management, University of Nigeria, Nsukka, Nigeria; 4 Department of Psychiatry and Mental Health, Unidade Local de Saúde de Loures-Odivelas, Loures, Portugal; 5 Department of Psychiatry and Mental Health, Unidade Local de Saúde de Lisboa Ocidental, Lisboa, Portugal; 6 Centro de Investigação Interdisciplinar em Saúde - CIIS, Faculdade de Medicina, Universidade Católica Portuguesa, Lisboa, Portugal; 7 Environmental Health Behaviour Lab, Instituto de Saúde Ambiental & Laboratório Associado TERRA, Faculdade de Medicina, Universidade de Lisboa, Lisboa, Portugal; 8 NOVA Medical School, Nova University of Lisbon, Lisbon, Portugal

**Keywords:** Mental health, Recovery, Support, Measurement, Reliability, Validity, INSPIRE

## Abstract

**Background:**

The importance of mental health services that support a recovery-oriented approach is increasingly recognized, and measures that evaluate this practice and promote change over time are needed. The INSPIRE measure is a 27-item questionnaire designed to assess service users’ perceptions of the support received from health professionals in their personal recovery. This study aimed to validate the Portuguese version of INSPIRE and assess its psychometric properties as a measure of staff support for personal recovery.

**Methods:**

The questionnaire survey was conducted from October 2023 to February 2024. Service users completed the Portuguese version of INSPIRE, the Client Satisfaction Questionnaire (CSQ-8), and a demographic and clinical questionnaire. Internal consistency was assessed using Cronbach’s alpha and McDonald’s model-based Omega. Test-retest reliability was assessed through the intraclass correlation coefficient (ICC) and weighted kappa. Convergent validity was examined by assessing correlation with CSQ-8. Factor validity was evaluated using exploratory factor analysis (EFA), with confirmatory factor analysis (CFA) performed to test the fit of factor structures derived from the EFA.

**Results:**

The study included 165 participants from seven psychosocial rehabilitation units which primarily target persons with a severe mental illness. For test–retest evaluation, 52 participants completed the questionnaire a second time. Internal consistency was satisfactory across all subscales and dimensions of the Support subscale, except for the Identity domain, which had marginally acceptable values. INSPIRE demonstrated significant positive correlations with CSQ-8 scores, supporting its convergent validity. EFA identified five factors for the Support scale and one factor for the Relationship scale, explaining 62% and 59% of the cumulative variance, respectively. CFA confirmed a good model fit for the Relationship scale and all Support subscales, except for the Identity and Empowerment subscales.

**Conclusions:**

The Portuguese version of INSPIRE showed strong internal consistency, as well as convergent and factor validity. This validated instrument can be applied in research and clinical settings to assess staff support for personal recovery and promote recovery-oriented mental health practices.

**Clinical trial number:**

Not applicable.

## Background

Globally, there is growing recognition of the need to provide mental health services that emphasize a recovery-oriented approach [1–4]. Personal recovery is described as a deeply individual and unique process, defined as “a way of living a satisfying, hopeful, and contributing life despite the limitations caused by illness” [5]. Although each person’s experience of recovery is unique, common themes include the significance of hope, taking personal responsibility, receiving support from others, engaging in meaningful activities, and developing a positive sense of identity [6]. To capture this, a conceptual framework has identified five key processes in personal recovery: Connectedness, Hope, Identity, Meaning, and Empowerment (the CHIME Framework) [7]. The lived experiences and perspectives of individuals with mental health challenges are central to fostering a recovery-oriented culture [8]. Recovery, as a concept, was developed by and for individuals with mental health issues to describe their own journeys and affirm their identity beyond the limitations of their diagnoses [8]. Personal recovery differs from clinical recovery, which is based on the medical model and focuses on the “remission of illness” as the goal of recovery [9].

Recovery-oriented mental health practices and service delivery represent an approach that emphasizes personal recovery in mental health care. Insights from global best practices have outlined four key domains in supporting recovery: supporting personally defined recovery, working relationships, organisational commitment and promoting citizenship [10]. This approach integrates the principles of self-determination and personalised care, emphasises hope, social inclusion, community participation, personal goal setting and self-management [11], and is guided by a set of underpinning values different from those that have traditionally informed mental health services [12]. A mental health service system that is guided by the rehabilitation and recovery perspective has a more complete understanding of the total impact of severe mental illness, with an emphasis on treating the consequences of the illness rather than just the illness per se, and on meeting the multiple residential, vocational, educational, and social needs and wants [5]. Current guidelines advocate for the adoption of recovery-oriented approaches by mental health teams and services for several reasons: research indicates they lead to improved outcomes [13], longitudinal studies have provided evidence that recovery is achievable [14], and they align with the lived experiences of individuals with mental health problems, reflecting what has supported their recovery journeys [15, 16]. Despite this, the practical implementation and integration of recovery principles into everyday practice remain limited [17].

Various tools have been developed to evaluate the recovery orientation of mental health services, including the Attitudes towards Recovery Questionnaire (ARQ) [18], INSPIRE [19], the Mental Health Recovery Measure (MHRM) [20, 21], the Provider Expectations for Recovery Scale (PERS) [22], the Recovery Attitudes Questionnaire (RAQ) [23], the Recovery Knowledge Inventory (RKI) [24], the Recovery Promoting Relationships Scale (RPRS) [25], the Recovery-Oriented Services Assessment (ROSA) [26], and RECOLLECT [27]. However, there is currently no single, universally accepted measure that meets all criteria: psychometric validity and reliability, sensitivity to change, ease of use, and compatibility with both the conceptual frameworks of personal recovery and recovery-oriented services or systems. This lack of a standardized tool presents an ongoing challenge. Among these, INSPIRE stands out as the only measurement tool that aligns closely with the CHIME framework, is validated, reliable, and capable of assessing the recovery orientation of services from the perspective of the service user [28, 29].

The full version of INSPIRE measure is a 27-item questionnaire designed to assess service users’ experiences of support from health professionals in their personal recovery [19]. It consists of two subscales: a 20-item Support subscale and a 7-item Relationship subscale. The Support subscale assesses the CHIME recovery processes, covering five domains: Connectedness (items S1–S4), Hope (items S5–S8), Identity (items S9–S12), Meaning and Purpose (items S13–S16), and Empowerment (items S17–S20). Each item is initially rated as being important for recovery (yes/no). For the items rated as important (i.e., yes), the level of support received from a mental health worker is rated on a 5-point Likert scale, ranging from “not at all” to “very much”. The Relationship subscale evaluates the working relationship between service users and providers, with items rated on a 5-point Likert scale from “strongly disagree” to “strongly agree.” Scores are calculated for each subscale, ranging from 0 (lowest support for recovery) to 100 (highest support for recovery). Scores below 72% in the Support subscale suggest the support may not be perceived as helpful, while scores below 78% in the Relationship subscale indicate that the quality of the relationship is perceived as insufficient and could be improved.

Standardized recovery measures play a critical role in advancing research in this area and developing recovery-oriented system. Validation involves testing instruments across diverse settings to determine their accuracy, dependability and consistency in measuring what it purports to measure [30]. In Portugal, we are now living a momentum of transformation and reform in mental health care, with the development of community mental health teams and recovery-oriented interventions and services. The validation of the full version of INSPIRE could inform clinical interventions, evaluation strategies, service development, and workforce planning. Additionally, using INSPIRE across different teams and over time could promote cross-team learning, guide policy decisions, and track progress in service improvement.

The INSPIRE tool has been validated among community mental health service users in Japan [31] and in Sweden [32], demonstrating its reliability and validity. The present study aims to assess the internal consistency, test-retest reliability, and convergent and factor validity of the Portuguese version of INSPIRE for users of mental health services in Portugal.

## Methods

### Study setting

This study was conducted in psychosocial rehabilitation units, including four community rehabilitation centers and three day-hospitals within public psychiatric departments, all situated in the Lisbon Metropolitan Area. These units primarily serve individuals diagnosed with severe mental illness. These services were chosen because they were already involved in a broader ongoing study. The survey was conducted from October 2023 to February 2024.

The authors took the questionnaires to the psychosocial rehabilitation units and information was provided to the staff to describe the INSPIRE measure and the study of evaluation of its psychometric properties and to explain the process of distributing and completing the questionnaire. Staff were instructed to distribute the questionnaire to all patients attending the services during the study period, explaining the purpose of the study, but refraining from helping to fill out the questionnaire. Staff also cooperated in the re-test to verify the test-retest reliability.

### Study participants

All patients who were receiving services in the included psychosocial rehabilitation units were asked to participate.

Service users should meet the following inclusion criteria: (1) use of community psychosocial rehabilitation centers and day hospitals, (2) age 18 years or older, (3) having a severe mental illness, (4) being able to give informed consent to participate in the study. After giving written informed consent, participants completed the questionnaire themselves. Participation was voluntary.

### Measures

#### Client satisfaction questionnaire

Service user satisfaction was measured using the 8-item Client Satisfaction Questionnaire (CSQ-8). Each item is rated on a 4-point Likert scale, ranging from “poor” to “excellent.” The total score can range from 8 to 32, with higher scores reflecting higher levels of satisfaction [33].

### Demographic and clinical variables

Demographic and clinical variables included age, gender, marital status, educational level, employment status, monthly income and psychiatric diagnosis.

### Procedure

After obtaining permission from the authors to translate and use INSPIRE, a bilingual native speaker with clinical experience translated the 27-item full English version into Portuguese. This translation was then reviewed by a team of expert native speakers. Following this, an independent translator performed a back-translation of the Portuguese version into English, which was subsequently verified for accuracy by a team of experts. The instrument was then pilot tested with 5 patients from one of the community psychosocial rehabilitation centers involved in the study to identify any issues or misunderstandings with the terminology. No adjustments were needed at this stage.

The study instrument was given to the participants at the initial time point (T0), and the same instrument was administered again two weeks later (T1). Participants completed the sociodemographic questionnaire at T0, and the 8-item Client Satisfaction Questionnaire (CSQ-8) was also recorded at T0.

The study received approval from the Ethical Committee of the Nova Medical School (nº 121/2023/CEFCM). Written informed consent was obtained from all participants.

### Statistical analysis

The data processing and statistical analyses for the study were conducted using R software (Version 4.1.3) [34] along with the dplyr (Version 1.1.0) and Psych (Version 2.2.3) packages. Descriptive statistics were initially performed to determine the number of valid responses for further analysis. Only responses with at least one completed item were included.

Reliability was assessed by estimating internal consistency for the support subscale as a whole and for each of its five domains, as well as for the relationship subscale, using Cronbach’s alpha [35] and McDonald’s model-based Omega (ω) [36]. A Cronbach’s alpha of 0.70 or higher was considered acceptable.

Test-retest reliability was evaluated in a subsample of respondents who completed the survey a second time two weeks later. Weighted Kappa (for nominal data) and intraclass correlation coefficients (ICC; for ordinal data) were calculated for each item to assess the agreement between the first and second assessments [37]. Kappa and ICC values were interpreted as follows: below 0.20 was poor agreement, 0.21–0.40 was fair, 0.41–0.60 was moderate, 0.61–0.80 was good, and 0.81–1.00 was very good agreement [37].

Convergent validity for the support and relationship subscales was evaluated by correlating them with the CSQ-8 using Pearson’s product-moment correlation. As there are no established criteria for convergent validity coefficients, values greater than 0.30 were considered satisfactory, reflecting a medium effect size according to Cohen [38].

For further analysis, factor validity was assessed for each of the two INSPIRE subscales using responses from participants who answered “yes” to all items in the support subscale (20 items) and the relationship subscale (7 items). The appropriateness of the data for exploratory factor analysis (EFA) was first tested using the Kaiser-Meyer-Olkin (KMO) measure of sampling adequacy and Bartlett’s test of sphericity, with a *p*-value less than 0.05 considered significant for each subscale. EFA for each subscale was conducted using oblimin rotation, and factor loadings greater than 0.3 were considered for interpretation. Confirmatory factor analysis (CFA) was then performed to assess how well the data fit the factor structure derived from the EFA. Based on theoretical assumptions, five underlying factors were hypothesized. Due to issues with matrix invertibility when Empowerment and Relationship were included, CFA was conducted separately for each latent variable. The chi-square fit index was used to evaluate the fit between the hypothesized model and the data [39], and the model fit was assessed using multiple indices: the ratio of χ2 to df (≤ 2), the Comparative Fit Index (CFI > 0.95), the Tucker-Lewis Index (TLI), and the Root Mean Square Error of Approximation (RMSEA < 0.07) [40, 41]. Two-tailed *p*-values below 0.05 were considered statistically significant.

## Results

### Sociodemographic characteristics of the study sample

A total of 165 questionnaires were collected with varying level of completion while 52 questionnaires were collected at the second assessment. A majority of the study sample were male (60.4%, *n* = 93), single (83.7%, *n* = 128) and had schizophrenia and other related diagnosis (60.7%, *n* = 91) (Table 1). The mean age of the study sample was 47.197 ± 12.329.

**Table 1:** Sociodemographic characteristics of the study sample

Variable	Frequency	Percentage^a^
Gender (*n* = 154)		
Male	93	60.4
Female	61	39.6
Marital status (*n* = 153)		
Single	128	83.7
Married	13	8.5
Divorced	10	6.5
Widowed	2	1.3
Education (*n* = 153)		
Primary	19	12.4
Basic	46	30.1
Secondary	59	38.6
University	29	19.0
Profession (*n* = 152)		
Low employed	12	7.9
Unemployed	46	30.3
Retired	50	32.9
Student	9	5.9
Domestic	1	0.7
Without activity	34	22.4
Monthly income (Euros) (*n* = 135)		
< 150	27	20.0
151–500	65	48.1
501–1000	32	23.7
1001–2000	8	5.9
> 2000	3	2.2
Diagnosis (*n* = 150)		
Schizophrenia & other related diagnosis	91	60.7
Depression	26	17.3
Bipolar disorder	15	10.0
Other ^b^	18	12.0

a: valid percentages are presented discounting missing variables. b: other include personality disorders, obsessive−compulsive disorder, and intellectual disability

### Reliability of the Portuguese-INSPIRE

The measures of internal consistency were found to be satisfactory while the test-retest reliability did not show good consistency between the two measurements as ICC values less than 0.5 are indicative of poor reliability. (Table 2)

**Table 2:** Reliability parameters of the Portuguese-INSPIRE

Internal consistency			Test-retest reliability	
Subscales	McDonald’s ω	Cronbach’s α	ICC	*p*-value
Support	0.93	0.93	0.023	0.33
Relationship	0.90	0.91	-0.011	0.65
Domains				
Connectedness	0.82	0.82	0.037	0.20
Hope	0.84	0.84	0.041	0.19
Identity	0.72	0.69	0.045	0.18
Meaning & Purpose	0.77	0.78	0.018	0.29
Empowerment	0.80	0.79	0.028	0.24

### Convergent validity

The correlation between the support subscale and the CSQ-8 score is positive and moderate (*r* = 0.47, *p*-value < 0.001) and correlation between the Relationship subscale and the CSQ-8 is also positive and moderate (*r* = 0.47, *p*-value < 0.001) demonstrating good convergent validity.

### Exploratory factor analysis

To evaluate the factor validity of the INSPIRE support subscale, 150 responses in which all 20 items in the subscale were answered “yes” were analysed, revealing five factors based on a loading cutoff criterion of 0.3. Similarly, for the relationship subscale, 155 responses where all 7 items were answered were used. The KMO score for the support subscale was 0.89, and Bartlett’s test of sphericity was significant (χ2 = 1892, df = 190, *p* < 0.001), indicating that factor analysis was appropriate. For the relationship subscale, the KMO score was 0.91, and Bartlett’s test of sphericity was also significant (χ2 = 612, df = 21, *p* < 0.001), confirming the suitability of factor analysis.

The first factor MR1 represents the “Hope” domain (S5 to S8). The third factor (MR3) is clearly representing the “Connectedness” domain (S1 to S4). Some items from “Meaning and purpose” (S13 to S16) and others from “Empowerment” (S17 to S20) are together in the MR4 factor, meaning that these two domains are not clearly distinct (Table 3). The factor structure obtained had a cumulative explained variance of 62% (MR1 = 19%; MR2 = 15%; MR3 = 13%; MR4 = 10%; MR 5 = 5%).

**Table 3:** Standardized factor loadings for the support subscale

	MR1	MR4	MR3	MR2	MR5
S1			0.85		
S2			0.492		
S3			0.628		
S4			0.588		
S5	0.818				
S6	0.604				
S7	0.388				0.336
S8	0.916				
S9		0.344			
S10	0.492	0.461			
S11				0.922	
S12				0.739	
S13		0.352			
S14	0.35	0.381			
S15		0.312			0.403
S16	0.405	0.422			
S17		0.652	0.302		
S18		0.815			
S19					0.314
S20				0.393	0.417

In the Factor Analysis dealing with Relationship items (*n* = 1 factor to extract), the factor loadings were large (> 0.70 in all cases) defining one Relationship factor (Table 4). The one-factor structure had a cumulative explained variance of 59%.

**Table 4:** Standardized factor loadings for the relationship subscale

	MR1	h2	u2
R1	0.82	0.68	0.32
R2	0.78	0.61	0.39
R3	0.72	0.52	0.48
R4	0.75	0.57	0.43
R5	0.78	0.61	0.39
R6	0.80	0.63	0.37
R7	0.71	0.50	0.50

### Confirmatory factor analysis

The R output could not compute the entire model because the main matrix was not invertible when Empowerment and Relationship domain were included. Excluding these domains yielded a model where the Chi-square had significant *p*-value (< 0.001), rejecting the null hypothesis that this model fits our data. So, a Confirmatory Factor Analysis was conducted for each latent variable.

Since the *p*-value of the Chi-square test is non-significant (*p* = 0.156) for Item 1–4 of the support scale, we can retain the null hypothesis that “Connectedness” is well represented by the 4 first questions of the questionnaire. For the “Hope” domain, the *p*-value (0.44) of the Chi-square test indicates that Hope is well represented by the questions S5 to S8. Considering the “Identity” domain S9 to S12, the low *p*-value (< 0.001) of the Chi-square test reveals that there is a mismatch between that factor and the data. For the Relationship feature, the *p*-value of the Chi-square test was non-significant (*p* = 0.284) when the scaled data was considered, meaning that Relationship subscale is well represented by the items R1 to R7 (Table 5).

**Table 5:** Fit indices for latent variables tested

Latent variable	χ2	Df	*p*-value	CFI	TLI	RMSEA
Connectedness (S1-S4)	3.714	6	0.156	0.975	0.926	0.076
Hope (S5-S8)	1.145	2	0.564	1.000	1.016	0
Identity (S9-S12)	21.296	2	< 0.001	0.746	0.238	0.254
Meaning & Purpose (S13-S16)	1.841	2	0.398	1	1.006	0
Empowerment (S17-S20)	35.456	2	< 0.001	0.830	0.491	0.334
Relationship (R1-R7)	16.492	14	0.284	0.991	0.986	0.034

χ2 = Chi-Square statistic; df = degrees of freedom; CFI = Comparative Fit Index; TLI = Tucker Lewis Index; RMSEA = Root Mean Square Error of Approximation

## Discussion

This study examined the psychometric properties of the Portuguese version of INSPIRE among users of psychosocial rehabilitation units, which primarily serve persons with a clinical diagnosis of severe mental illness. The findings indicate that INSPIRE demonstrates satisfactory internal consistency; however, the test-retest reliability did not exhibit strong consistency.

Participants were recruited from various psychosocial rehabilitation units, including community rehabilitation centers, day hospitals, and multiple clinical teams, providing a broad spectrum of service experiences and clinical backgrounds. The sample reflected demographic diversity in age, gender, socioeconomic status, and urban or rural residence, which may enhance the generalizability of the findings. Portugal’s linguistic uniformity, with no significant regional dialects, supports the assumption that the questionnaire would be consistently interpreted and representative of the national population. Additionally, the Lisbon metropolitan area attracts individuals from across the country, further strengthening sample representativeness. Given the national guidelines for mental health service organization, participants’ experiences are likely to reflect the broader national context.

Reliability refers to the consistency of results when a testing procedure is repeated [42]. There are various types of scale reliability: (1) the equivalence of items in a test (internal consistency) or of assessments made by different observers using the same instrument (interrater reliability) and (2) the stability of measurements taken at different times from the same individuals (test–retest reliability) [43].

Internal consistency evaluates how well all items in a scale measure the same construct [43]. It is recommended that new instruments achieve a minimum Cronbach’s alpha of 0.70, while established instruments should meet a threshold of 0.80 [19, 31, 32, 44]. In this study, both Cronbach’s alpha and McDonald’s omega indicated satisfactory internal consistency for the subscales and for the dimensions within the support subscale, except for the Identity domain, which showed marginally acceptable values. Like previous studies [19, 31, 32], the alpha coefficient for the total score of the support subscale was notably high (0,93), suggesting potential redundancy, with some items possibly addressing the same concepts in slightly different ways [44]. Future research with other Portuguese samples could explore whether reducing the number of items improves the instrument’s efficiency.

Test–retest reliability, which assesses the stability of measurements over time, is determined by administering the test at two different points in time to the same individuals and analysing the correlation or strength of association between the scores [43]. The timing of the second administration is critical: the interval should be long enough to avoid carryover effects from the initial assessment but short enough to prevent changes in health status or learning from altering responses [43]. The changing nature over time of the construct being measured is the second main factor which may influence the evaluation of test-retest reliability [45]. ICC (Intraclass Correlation Coefficient) is a reliability measure that assesses both the correlation and agreement between measurements, and values less than 0.5, between 0.5 and 0.75, between 0.75 and 0.9, and greater than 0.90 are indicative of poor, moderate, good, and excellent reliability, respectively [46]. In this study, the test-retest reliability showed inconsistent results. This result may be attributed to variations in the timing of the second assessment, which may have varied somewhat between the different psychosocial rehabilitation units. It may also reflect changes in participants’ functioning, mood or willingness to collaborate.

Validity assesses the extent to which an instrument measures what it is intended to measure [43]. Validity is not an inherent characteristic of the instrument itself but depends on its use in a particular context [42]. When an instrument is used in a new context, its validity must be evaluated [42]. In this study, correlations between INSPIRE subscales and the CSQ-8 were positive and moderate (Pearson’s correlation: 0.47), supporting INSPIRE’s validity as a measure of staff support for personal recovery.

Factor analysis explores relationships among survey items to determine whether subsets of items are more closely related to one another than to others, thereby analysing dimensionality among the items [42]. The exploratory factor analysis in this study identified five factors, but there was cross-loading of items in the Meaning and Purpose and Empowerment domains, suggesting that these items may measure overlapping concepts. This could indicate that Meaning and Purpose are not entirely distinct from Empowerment within the context of recovery. In fact, within the CHIME framework of mental health recovery, Meaning and Purpose and Empowerment are closely intertwined rather than entirely distinct concepts. Both components center around the individual’s agency and sense of self in the recovery journey. Meaning and Purpose involves having direction, goals, and a sense that life holds value, while Empowerment is about gaining confidence, autonomy, and control over one’s life. However, discovering meaning in one’s experiences, roles, or future aspirations often leads to a sense of empowerment. Conversely, feeling empowered can motivate someone to pursue or reclaim purpose in life. Rather than existing in isolation, these elements reinforce one another. Recognizing this interconnection is vital for person-centered recovery approaches, as it allows for more integrated and holistic support strategies. Confirmatory factor analysis of each latent variable revealed good model fit for most domains, although the Identity and Empowerment domains showed some inconsistencies. Certain items, such as “Feeling I can deal with stigma” (S9), may reflect concepts (stigma, in this case) that are more research-oriented and less familiar in everyday use. Additionally, items like “Having my spiritual belief respected” (S11) and “Having my ethnic/cultural/racial identity respected” (S12), also within the Identity domain, may reflect cultural and ethnic differences between the British population, where the INSPIRE instrument was originally developed, and the population that participated in the validation of the instrument in Portugal, with less ethnic diversity. These variations might also stem from the heterogeneity of the studied sample or non-independent observations [47].

For the Relationship subscale, factor loadings of 0.70 or higher confirmed the one-factor structure consistent with the structure of the original INSPIRE [19]. The Confirmatory factor analysis also indicated good model fit with satisfactory fit indices.

### Strengths and limitations

This study makes a valuable contribution by validating a measure to assess staff support for personal recovery within the Portuguese context. Participants were a convenience sample of service users from various psychosocial rehabilitation units in the Lisbon metropolitan area. Nevertheless, some methodological limitations should be noted. First, the selection of participants from specific geographic areas and service types may restrict the generalizability of the findings. However, the sample reflects clinical and demographic diversity in age, gender, socioeconomic status, urban/rural residence, and service background. Moreover, Portugal’s relative linguistic and organizational homogeneity supports broader applicability. Second, the test-retest reliability revealed limited consistency. Future research should adopt a more rigorous approach to timing between assessments to better evaluate this dimension. Third, convergent validity was assessed using the Portuguese version of the Client Satisfaction Questionnaire, even though this tool has not been previously validated. Fourth, the rarity of service users evaluating psychiatric service quality, service providers, and recovery processes adds complexity to the study design and interpretation. Finally, the questionnaires were distributed by the staff under evaluation. Although anonymity and confidentiality were assured, this distribution method may have introduced bias.

## Conclusions

This study confirmed the internal consistency, as well as the convergent and factor validity, of the Portuguese version of INSPIRE among users of psychosocial rehabilitation units in Portugal. The findings support INSPIRE as a reliable tool for assessing staff support for personal recovery. By facilitating structured conversations between users and staff, INSPIRE can promote person-centered care and help identify individual support priorities. It also offers a valuable means of evaluating personal recovery experiences, comparing practices against national and international standards, and monitoring progress over time — contributing to the advancement of a recovery-oriented mental health system. Furthermore, INSPIRE may play a key role in developing training programs for mental health professionals and guiding future research on how services can better support personal recovery in Portuguese-speaking countries.

## Data Availability

The datasets generated and analysed during the current study are available from the corresponding author on reasonable request.

## Abbreviations

ARQ: Attitudes towards Recovery Questionnaire
CFA: Confirmatory factor analysis
CFI: Comparative fit index
CHIME: Connectedness, hope, identity, meaning and empowerment
CSQ-8: Client satisfaction questionnaire
EFA: Exploratory factor analysis
ICC: Intraclass correlation coefficients
KMO: Kaiser-meyer-olkin
MHRM: Mental health recovery measure
PERS: Provider expectations for recovery scale
RAQ: Recovery attitudes questionnaire
RKI: Recovery knowledge inventory
RMSEA: Root mean square error of approximation
RPRS: Recovery promoting relationships scale
ROSA: Recovery-oriented services assessment
TLI: Tucker lewis index
